# Bacteriological profile and antimicrobial sensitivity pattern of blood culture isolates among septicemia-suspected children at Tikur Anbessa Specialized Hospital and Yekatit 12 Hospital, Addis Ababa, Ethiopia

**DOI:** 10.1186/cc12911

**Published:** 2013-11-05

**Authors:** Adugna Negussie, Gebru Mulugeta, Ahmed Bedru, Ibrahim Ali, Damte Shimeles, Tsehaynesh Lema, Abraham Aseffa

**Affiliations:** 1 Department of Medical Laboratory Science, College of Health Science, Addis Ababa University, Ethiopia; 2 Department of Public Health Officer, Jigjiga University, Jigjiga, Ethiopia; 3 Armauer Hansen Research Institute, Addis Ababa, Ethiopia; 4 Tikur Anbessa Specialized Hospital, Department of Pediatrics, Addis Ababa, Ethiopia

## Background

Septicemia is a systemic disease caused by the spread of microorganisms and their toxins in the blood. These bloodstream infections are a major cause of morbidity and mortality in children in developing country [1-4]. It has been confirmed by culture that is associated with clinical manifestation and systemic response [5-7]. It is crucial to continuously monitor any change in the local patterns of infection and susceptibility to various antibiotics. The aim of this study was to determine the bacteriological profile and antimicrobial sensitivity patterns among children suspected of having septicemia.

## Materials and methods

A cross-sectional study involved about 201 pediatric patients (≤12 years) was conducted from October 2011 to February 2012 at Tikur Anbessa Specialized Hospital and Yekatit 12 Hospital's pediatric units after the proposal of this study was approved by National Ethics Review Committee. Standard procedure was followed for blood sample collection. Samples were incubated in the BACTEC 9050 System, followed by isolate identifications based on standard microbiological procedures and testing for their susceptibility to antimicrobial agents using the disc diffusion method. Data were analyzed using the SPSS version 19 software package.

## Results

**Figure 1:**
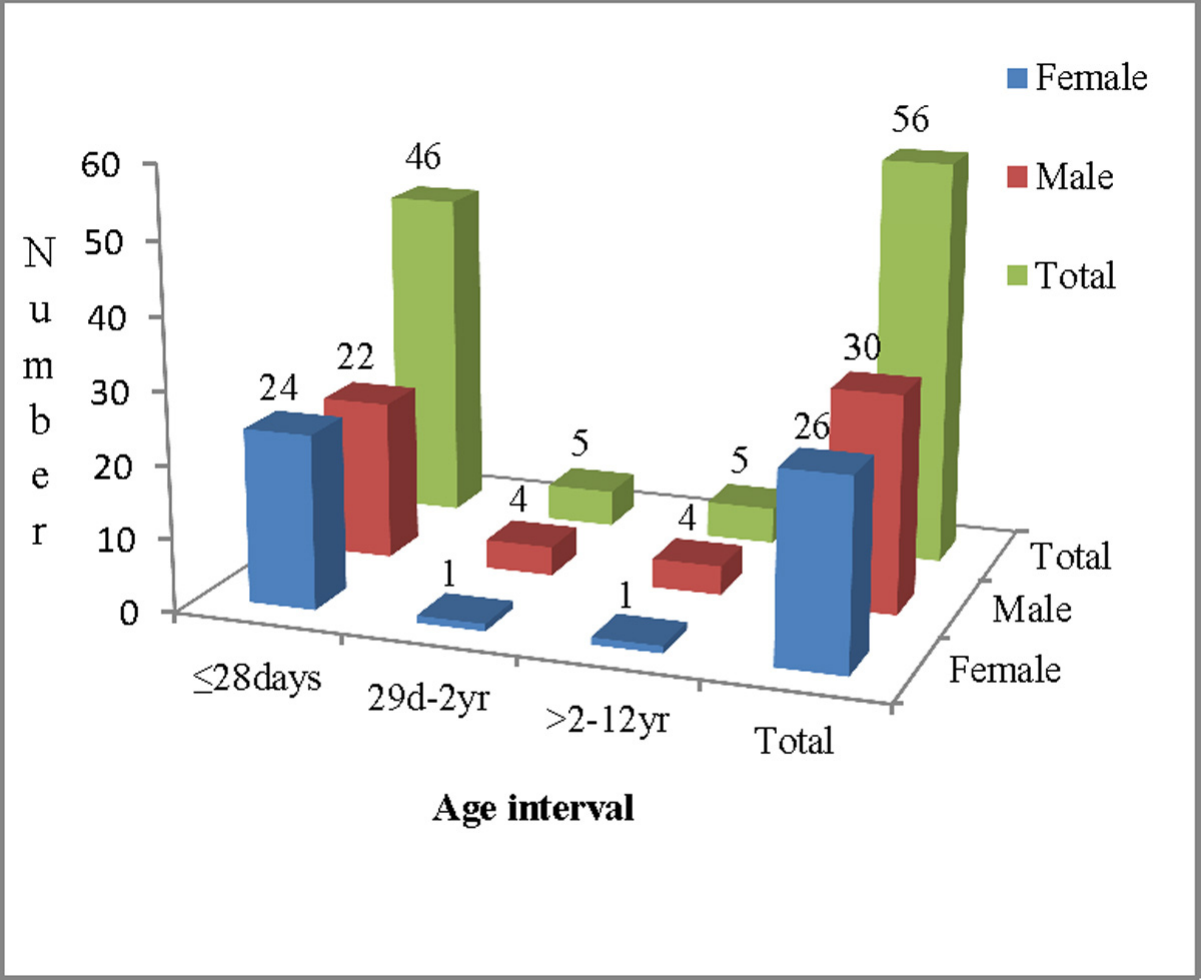
**Distribution of 56 blood culture isolates by age interval and gender**.

**Figure 2:**
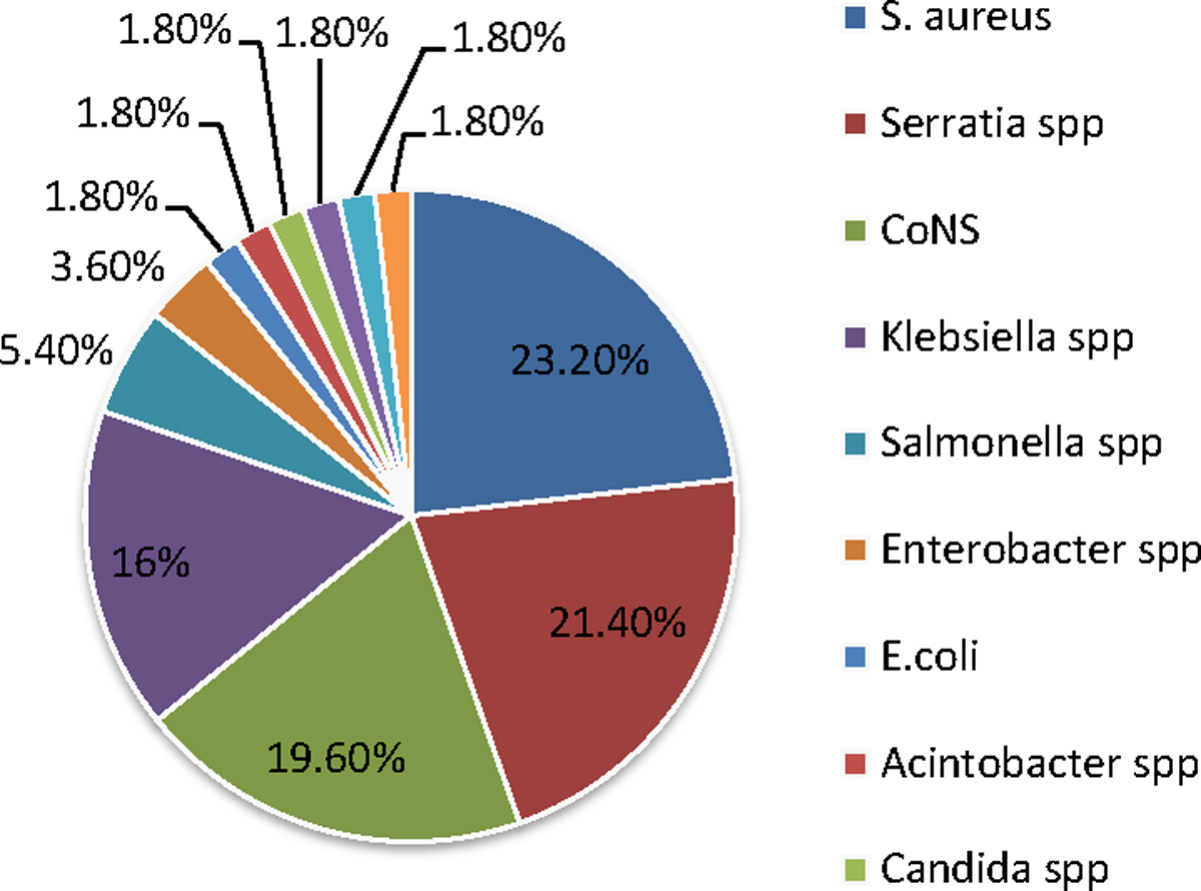
**Distribution of blood culture isolates in children with suspected of having sepsis**.

Out of 201 study subjects, 110 (54.7%) were male. The majority (147, 73.1%) of them were neonates (≤28 days). The mean length of hospitalization was 11.24 days. Out of the 201 tested blood samples, blood cultures were positive in 56 (27.9%) cases (Figure 1). Gram-negative and Gram-positive bacteria constituted 51.8% and 46.4%, respectively. The most frequent pathogen found was *Staphylococcus aureus *(23.2%), followed by *Serratia marcescens *(21.4%), CoNS (19.6%), *Klebsiella *spp. (16%), *Salmonella *spp. (5.4%) and *Enterobacter cloacae *(3.6%) (Figure 2). The majority of bacterial isolates showed high resistance to ampicillin, penicillin, co-trimoxazole, gentamicin and tetracycline. Ciprofloxacin and nalidixic acid were the most effective antimicrobial agents for Gram-negative bacteria, while vancomycin and clindamycin for Gram-positive bacteria (Table 1). Deaths occurred in 25 (12.4%) children, out of which 13 (23.2%) had bacteremia.

**Table 1:** Antimicrobial resistance pattern of bacteria isolated from blood culture

Antimicrobial drugs	Resistance proportion of bacterial isolates (R%)							
	
	*S. aureus *(*n *= 13)	*S. marcescens *(*n *= 12)	CoNS (*n *= 11)	*Klebsiella *spp. (*n *= 9)	*Salmonella *spp. (*n *= 3)	*E. cloacae *(*n *= 2)	Other GNB^a ^(*n *= 3)	Other GPB^b ^(*n *= 2)
Penicillin	92.3	ND	81.8	ND	ND	ND	ND	100
Ampicillin	84.6	100	100	100	100	100	100	100
Clindamycin	0	ND	0	ND	ND	ND	ND	0
Erythromycin	30.8	ND	54.5	ND	ND	ND	ND	0
Chloramph.	7.7	25	36.4	44.4	100	0	100	50
Ciprofloxacin	30.8	0	18.2	44.4	0	0	0	0
Vancomycin	15.4	ND	18.2	ND	ND	ND	ND	0
Cefoxitin	38.5	33.3	54.5	0	0	0	66.7	50
Gentamycin	30.8	91.7	36.4	100	100	0	100	50
Tetracycline	53.8	91.7	45.5	55.5	100	100	66.7	100
SXT	61.5	91.7	81.8	77.8	100	100	66.7	0
Ceftriaxone	46.2	16.7	27.3	100	100	100	100	50
Nalidixic acid	ND	0	ND	44.4	0	0	0	ND

ND, not done; SXT, sulphamethaxozol/trimethoprim. ^a^Gram-negative bacteria (*Acinetobacter baumanii, Escherichia coli*, and *Pseudomonas leutole*). ^b^Gram-positive bacteria (*Enterococcus *spp. and *Streptococcus*. spp.).

## Conclusions

The present study revealed that both Gram-positive and Gram-negative bacteria were responsible for bloodstream infections and the majority of the isolates were multidrug resistant. *S. aureus *and *S. marcescens *were the most common isolated bacteria from blood cultures. The alarmingly higher percentages of multidrug-resistant isolates urge us to take infection prevention measures and to conduct other large studies for appropriate empiric antibiotic choice.
